# Physiopathological Role of the Vesicular Nucleotide Transporter (VNUT) in the Central Nervous System: Relevance of the Vesicular Nucleotide Release as a Potential Therapeutic Target

**DOI:** 10.3389/fncel.2019.00224

**Published:** 2019-05-17

**Authors:** María T. Miras-Portugal, Aida Menéndez-Méndez, Rosa Gómez-Villafuertes, Felipe Ortega, Esmerilda G. Delicado, Raquel Pérez-Sen, Javier Gualix

**Affiliations:** ^1^Departamento de Bioquímica y Biología Molecular, Facultad de Veterinaria, Universidad Complutense de Madrid, Madrid, Spain; ^2^Instituto Universitario de Investigación en Neuroquímica, Universidad Complutense de Madrid, Madrid, Spain; ^3^Instituto de Investigación Sanitaria del Hospital Clínico San Carlos, Madrid, Spain

**Keywords:** VNUT, vesicular ATP release, purinergic signaling, neuropathic pain, glaucoma, clodronate

## Abstract

Vesicular storage of neurotransmitters, which allows their subsequent exocytotic release, is essential for chemical transmission in the central nervous system. Neurotransmitter uptake into secretory vesicles is carried out by vesicular transporters, which use the electrochemical proton gradient generated by a vacuolar H^+^-ATPase to drive neurotransmitter vesicular accumulation. ATP and other nucleotides are relevant extracellular signaling molecules that participate in a variety of biological processes. Although the active transport of nucleotides into secretory vesicles has been characterized from the pharmacological and biochemical point of view, the protein responsible for such vesicular accumulation remained unidentified for some time. In 2008, the human *SLC17A9* gene, the last identified member of the SLC17 transporters, was found to encode the vesicular nucleotide transporter (VNUT). VNUT is expressed in various ATP-secreting cells and is able to transport a wide variety of nucleotides in a vesicular membrane potential-dependent manner. VNUT knockout mice lack vesicular storage and release of ATP, resulting in blockage of the purinergic transmission. This review summarizes the current studies on VNUT and analyzes the physiological relevance of the vesicular nucleotide transport in the central nervous system. The possible role of VNUT in the development of some pathological processes, such as chronic neuropathic pain or glaucoma is also discussed. The putative involvement of VNUT in these pathologies raises the possibility of the use of VNUT inhibitors for therapeutic purposes.

## Introduction

Chemical transmission at the synapse plays a central role in cellular communication in both the central and peripheral nervous system. This process requires the previous storage of neurotransmitters into synaptic vesicles and their subsequent release through Ca^2+^-dependent exocytosis. The released neurotransmitters interact with specific receptors on the plasma membrane of target cells, thus triggering intracellular signals. Vesicular neurotransmitter transporters (VNTs) are the proteins responsible for the storage of these compounds into synaptic vesicles, thus determining the amount of neurotransmitter available to be released by exocytosis and, therefore, are essential components of chemical transmission in the nervous system (Blakely and Edwards, 2012; Omote and Moriyama, 2013; Omote et al., 2016). Different types of transporters are known to be involved in the uptake of neurotransmitters into synaptic vesicles. These include the vesicular glutamate transporters (VGLUTs), vesicular monoamine transporters (VMATs), vesicular acetylcholine transporter (VAChT), and vesicular inhibitory amino acid transporter (VIAAT) (Eiden et al., 2004; Gasnier, 2004; Reimer, 2013). These vesicular transporters are active transport systems that mediate the accumulation of their respective neurotransmitters by means of an electrochemical proton gradient (ΔμH^+^) across the vesicular membrane, which is generated by a vacuolar H^+^-ATPase (V-ATPase) (Njus et al., 1986; Whittaker, 1987). ΔμH^+^ is composed of a proton gradient (ΔpH, lumen acidic) and the membrane potential (ΔΨ, lumen-positive). Vesicular neurotransmitter transporters use the chemical (ΔpH), electrical (ΔΨ) or both components of ΔμH^+^ as driving forces to mediate transport of neurotransmitters against their concentration gradient (Chaudhry et al., 2008).

ATP is a relevant chemical transmitter released from neurons and non-neuronal cells. In the extracellular space, this nucleotide undergoes successive hydrolysis by ectonucleotidases and both ATP and some of its enzymatic breakdown products (ADP and adenosine) can interact with specific cell-surface purinoceptors (Burnstock, 2007b). In the nervous system, purinergic signaling is involved in a great variety of either physiological or pathological processes such as mechanosensory transduction, central control of autonomic functions, glia-glia and neuronal-glial interaction, pain, trauma, ischemia or inflammation. In addition, extracellular nucleotides exert potent long-term effects in cell proliferation, growth and development (Burnstock, 2007a; Abbracchio et al., 2009; Burnstock et al., 2011). In spite of the relatively well-known features of the signaling cascade after ATP secretion and activation of the purinergic receptors, the mechanism by which ATP is released from cells remains less understood. There is compelling evidence for neuronal exocytotic release of ATP (Richardson and Brown, 1987; Sawynok et al., 1993; Jo and Schlichter, 1999; Pankratov et al., 2006, 2007; Tompkins and Parsons, 2006) and studies also support a vesicular release of ATP from glial cells (Pascual et al., 2005; Pankratov et al., 2006; Bowser and Khakh, 2007; Pangrsic et al., 2007; Zhang et al., 2007; Parpura and Zorec, 2010; Lalo et al., 2014), although additional mechanisms of nucleotide release through ATP-binding cassette transporters, pannexin or connexin hemichannels, voltage-dependent anion channels or P2X7 receptors cannot be ruled out (Lazarowski, 2012; Baroja-Mazo et al., 2013). Consistent with this, the determination of the mechanism and physiological relevance of vesicular ATP storage and its exocytotic release, that is, vesicular ATP release, appear to be critical for the full understanding of purinergic chemical transmission at the nervous system.

**FIGURE 1:**
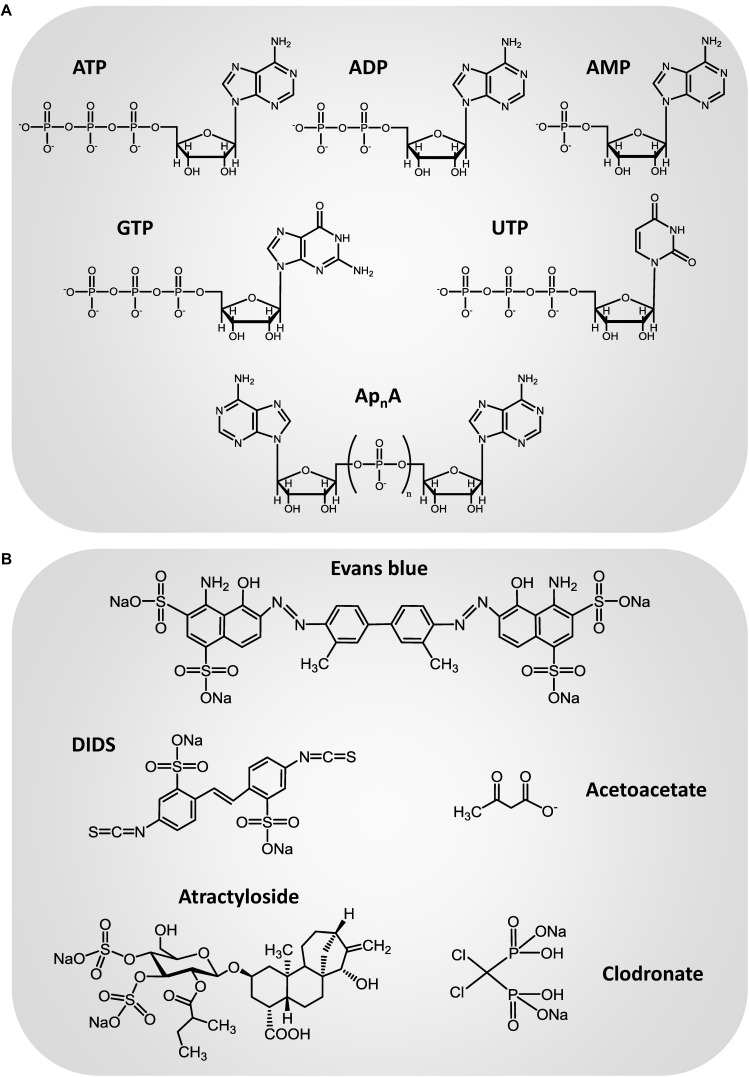
Chemical structure of the main transport substrates **(A)** and inhibitors **(B)** of the vesicular nucleotide transporter.

In the past decades, it has been recognized that ATP is a common constituent of secretory vesicles (Winkler, 1976; Holmsen and Weiss, 1979; Hutton et al., 1983; Njus et al., 1986; Johnson, 1988). The presence of an active transport mechanism that mediate nucleotide accumulation in a ΔΨ-dependent manner has been described in adrenal medulla chromaffin granules and the kinetic behavior, solute specificity and pharmacology of the nucleotide vesicular transporter have been extensively analyzed in this secretory vesicle model (Kostron et al., 1977; Aberer et al., 1978; Weber and Winkler, 1981; Bankston and Guidotti, 1996; Gualix et al., 1996, 1997, 1999a). When transport was assayed using a wide range of substrate concentrations, complex non-hyperbolic saturation curves were obtained. This complex dependence of transport capacity with substrate concentration was explained on the basis of a mnemonical kinetic model (Gualix et al., 1996, 1997). The mnemonic kinetic behavior of the nucleotide vesicular transporter was further corroborated by flow cytometry analysis of fluorescent nucleotide analogs incorporation into single chromaffin granules (Gualix et al., 1999a). Another remarkable feature of the nucleotide vesicular transporter in chromaffin granules is its low specificity (Aberer et al., 1978; Weber and Winkler, 1981; Bankston and Guidotti, 1996; Gualix et al., 1996, 1997), this transporter being able to internalize a wide diversity of nucleotides such as ATP, ADP, AMP, GTP, and UTP (Figure 1A), as well as the diadenosine polyphosphates (Ap_n_A), a group of dinucleotides that are also constituents of secretory vesicles (Rodriguez del Castillo et al., 1988; Pintor et al., 1992a,b,c) and can be detected in the extracellular media both under basal conditions (Gualix et al., 2014) or after stimulation of the cells with depolarizing agents or secretagogues (Pintor et al., 1992a,c). Nucleotide uptake into chromaffin granules is significantly reduced in the presence of atractyloside (Figure 1B), a compound previously described as an inhibitor of the mitochondrial ADP/ATP exchanger, and the anion transport inhibitor 4,4′-diisothiocyanatostilbene-2,2′-disulfonic acid (DIDS) (Kostron et al., 1977; Aberer et al., 1978; Weber and Winkler, 1981; Bankston and Guidotti, 1996; Gualix et al., 1996, 1997). A nucleotide vesicular transporter with strong similarities to that described in chromaffin granules can be also observed in cholinergic synaptic vesicles isolated from *Torpedo marmorata* electric organ and brain synaptic vesicles. Again, several different nucleotides can be incorporated into the vesicles through this vesicular transporter (Luqmani, 1981; Gualix et al., 1999b), which also exhibits a complex kinetic behavior that can be explained by means of a mnemonical model (Gualix et al., 1999b). As occurs in chromaffin granules, nucleotide transport into synaptic vesicles is affected by atractyloside and DIDS, although the compound that showed more effectivity to reduce ATP uptake into the vesicles was Evans blue (Figure 1B; Gualix et al., 1999b), a dye which was previously described as a potent inhibitor of the vesicular glutamate transporter (Roseth et al., 1995). However, in spite of the accumulating evidence of the presence of an active mechanism of nucleotide transport into secretory vesicles, such as synaptic vesicles and adrenal chromaffin granules, the protein responsible for the nucleotide vesicular accumulation remained unidentified for some time.

Solute carrier 17 (SLC17) type I inorganic phosphate transporters are a group of structurally related proteins that mediate the transmembrane transport of organic anions. Members of this transporter family include the three identified isoforms of the vesicular glutamate transporter VGLUT1 (*SLC17A7*), VGLUT2 (*SLC17A6*), and VGLUT3 (*SLC17A8*) (Reimer, 2013). In 2008, *SLC17A9*, the last identified member of this family was found to encode the vesicular nucleotide transporter (VNUT). Northern blot analysis revealed that *SLC17A9* gene is expressed in several organs, being especially abundant in the brain and the adrenal gland, regions where nucleotide vesicular transport might be relevant (Sawada et al., 2008). Moreover, in the adrenal gland, the SLC17A9 protein is specifically expressed in the medulla, where it is associated with chromaffin granules, as revealed by immunohistochemistry, immunoelectron microscopy and Western blot (Sawada et al., 2008). These data are compatible with the hypothesis that SLC17A9 could be responsible for the granular storage of the nucleotides. The human SLC17A9 protein was expressed, purified and incorporated into liposomes and an internal positive Δψ was generated by diffusion of K^+^ into the liposomes by the addition of valinomycin, in order to supply a driving force for nucleotide uptake. As anticipated, the SLC17A9 protein actively transported ATP at the expense of Δψ but not of ΔpH. This protein carried several nucleotides with the following order of efficacy: ATP > UTP > GTP > ITP, ADP > > AMP. Adenosine cannot be transported by the SLC17A9 protein, whereas the diadenosine polyphosphate Ap_3_A is a good transport substrate. The substrate selectivity of the SLC17A9 protein approximately matches the nucleotide content of the organelles that store ATP (Sawada et al., 2008). Additionally, SLC17A9 does not transport substrates of other members of the SLC17 family such as glutamate, aspartate, or hippuric acid, which indicates that the SLC17A9 protein is a carrier that specifically recognizes a variety of nucleotides as transport substrates. SLC17A9-mediated ATP transport is inhibited by DIDS and Evans blue (Sawada et al., 2008). Atractyloside is also a partial inhibitor but only when Mg^2+^ is present in the assay medium. These characteristics are similar, if not identical, to those observed for the transport of nucleotides to chromaffin granules and synaptic vesicles. Moreover, SLC17A9 protein is endogenously expressed by PC12 pheochromocytoma cells, where it is associated with secretory granules. Suppression of SLC17A9 gene expression by RNA interference (RNAi) strongly decreased vesicular storage and release of ATP from PC12 cells. Taken together, all these results demonstrate that *SLC17A9* encodes VNUT (Sawada et al., 2008; Moriyama et al., 2017). When mouse VNUT protein was purified and reconstituted in liposomes (Larsson et al., 2012) it exhibited similar functional properties to the previously characterized human orthologue (Sawada et al., 2008), thus indicating that *SLC17A9* also encodes the VNUT in rodents. An additional evidence of the essential role of this protein in the vesicular storage of nucleotides is that the vesicular release of ATP is lost from neurons and neuroendocrine cells of *VNUT* knockout (*VNUT^-/-^*) mice (Sakamoto et al., 2014; Masuda et al., 2016; Nakagomi et al., 2016; Moriyama et al., 2017).

## VNUT Distribution in Neuronal and GLIAL Populations of the Central Nervous System

### Expression and Distribution of VNUT in the Brain

The seminal work of Sawada et al. (2008) showed the expression of VNUT in human and mouse brain but its cellular and subcellular distribution was not known.

**FIGURE 2:**
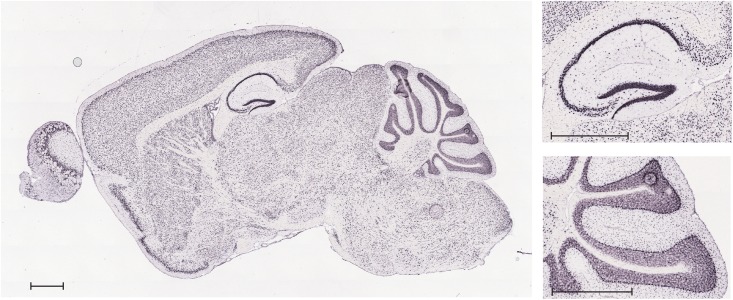
*In situ* hybridization analysis of VNUT transcript expression in a sagittal section of adult mouse brain. Inserts show magnification of the hippocampal and cerebellar areas. Scale bar: 850 μm. Image credit: Allen Mouse Brain Atlas (http://mouse.brain-map.org/gene/show/86822). Image is reproduced with permission of the copyright holders.

Allen Mouse Brain Atlas provides a comprehensive atlas of gene expression in the adult C57BL/6J mouse brain. Data were generated using automated high-throughput procedures for *in situ* hybridization and data acquisition (Lein et al., 2007), and are publicly accessible online^1^. As shown in Figure 2, VNUT transcript is widely expressed throughout the adult mouse brain, with a prominent expression found in areas such as the hippocampus or the cerebellum.

Immunoperoxidase labeling of rat brain tissue also showed that VNUT is widely distributed throughout the brain with particular strong VNUT immunoreactivity in the cerebellum, the hippocampus and the olfactory bulb (Larsson et al., 2012).

#### VNUT in the Hippocampus

**FIGURE 3:**
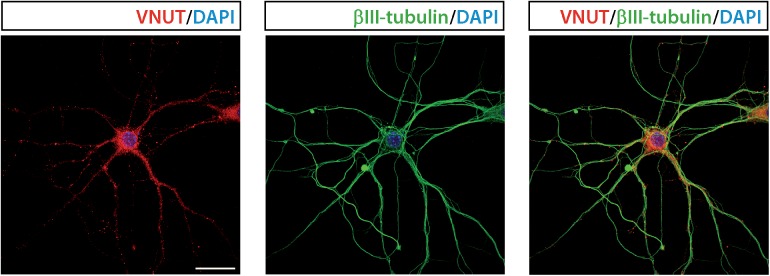
Vesicular nucleotide transporter is expressed by cultured hippocampal neurons. Representative immunofluorescence images showing immunostaining for VNUT (red) and the cytoskeletal protein βIII-tubulin (green) in cultured hippocampal neurons at 7 days *in vitro*. The nuclei are counterstained with DAPI (blue). Scale bar: 20 μm. Adapted from Menéndez Méndez (2017). Images are reproduced with permission of the copyright holders.

Figure 3 shows immunofluorescence images demonstrating the presence of VNUT in cultured mouse hippocampal neurons at 7 days *in vitro*.

Vesicular nucleotide transporter immunofluorescence could be detected in the soma and neurites of cultured hippocampal neurons, which also showed VNUT-dependent ATP release, as K^+^-evoked ATP release was attenuated by RNAi-mediated knockdown of VNUT (Larsson et al., 2012). The staining pattern of VNUT in the hippocampal neurons only partially overlapped with the presynaptic terminal markers synaptophysin and synaptotagmin 1, thus indicating that VNUT was located in different neuronal compartments. The analysis of the subcellular localization of VNUT in the hippocampal neuropil by immunogold labeling and electron microscopy showed that the nucleotide transporter is associated with synaptic vesicles in both excitatory and inhibitory terminals (Larsson et al., 2012). Moreover, VNUT immunoreactivity can be also detected in preterminal axons, where it was mainly located to the axoplasm as opposed to near the plasma membrane, indicating that VNUT-possessing vesicles are undergoing axonal transport (Larsson et al., 2012). VNUT also appears to be associated with vesicular structures in postsynaptic dendritic spines in the hippocampal formation. Although the role of the postsynaptically localized VNUT have to be clarified in further studies, authors hypothesize that postsynaptic spines can be an additional potential source of extracellular ATP, which may act as a retrograde signal, thus modulating presynaptic transmitter release (Larsson et al., 2012). Immunoisolation experiments showed that some, but not all, VGLUT1-possessing synaptic vesicles also contain VNUT, thus indicating that ATP is stored only in a subpopulation of glutamatergic vesicles. Analysis of immunogold labeling in hippocampal inhibitory terminals suggested that a similar partial segregation exists between VNUT- and VGAT-possessing vesicles (Larsson et al., 2012). Taken together, all these data indicate that VNUT may confer a purinergic phenotype to hippocampal neurons by establishing an exocytotically releasable vesicular pool of ATP. Hippocampal neurons from *VNUT^-/-^* mice completely loss their capacity to release ATP in response to K^+^ stimulation (Sakamoto et al., 2014), which is an additional confirmation of the essential role of VNUT in the neuronal vesicular storage and vesicular release of ATP in the hippocampus.

#### VNUT in the Cerebellum

When localization of VNUT was analyzed by immunoperoxidase labeling in the rat brain, particularly strong immunoreactivity was observed in the cerebellar cortex. VNUT immunolabeling was detected in the somatodendritic extent of Purkinje cells, being also relevant throughout the molecular layer (Larsson et al., 2012).

Further studies have shown that cultured cerebellar granule neurons express a functional VNUT that participates in the exocytotic release of ATP from these cells (Menendez-Mendez et al., 2017). VNUT can be detected by western blotting and immunofluorescence in the cultured granule neurons, which release ATP in a Ca^2+^-dependent manner, as stimulation of the cells with the calcium-selective ionophore ionomycin induces a significant increase of extracellular ATP (Menendez-Mendez et al., 2017). Exocytosis of ATP-containing vesicles can be visualized by fluorescence microscopy using quinacrine. This acidophilic antimalarial drug interacts with ATP stored in vesicles and has been extensively used as a fluorescent marker of intracellular ATP storage sites (Orriss et al., 2009; Liu et al., 2016). When granule cells were loaded with quinacrine, a punctate staining was observed, which appeared not only throughout the soma but was also evident in cell prolongations, thus indicating the presence of numerous ATP enriched vesicles in the granule neurons. Depolarization of the cells with K^+^ reduced quinacrine-associated fluorescence in granule cells, showing that they release ATP via Ca^2+^-dependent exocytosis (Menendez-Mendez et al., 2017). Ionomicin-induced ATP release was reduced when granule neurons were treated with the VNUT inhibitor Evans blue, thus indicating the involvement of VNUT in the vesicular storage and release of ATP. Moreover, immunofluorescence assays showed the co-localization of the vesicular protein synaptophysin and VNUT immunostaining in granule neurons, which further supports the exocytotic nature of ATP release (Menendez-Mendez et al., 2017). However, co-localization of VNUT and the synaptic vesicle marker was incomplete, suggesting that VNUT may be also present in another type of storage vesicles or subcellular structures. The subcellular distribution of VNUT in cerebellar granule neurons was analyzed by the use of specific axonal and somatodendritic markers, such as the pan-axonal neurofilament marker SMI 312 and the microtubule-associated protein 2 (MAP2), respectively. VNUT showed co-localization with both subcellular markers, suggesting that this transporter exists both pre- and post-synaptically in the granule cells. The presence of VNUT in postsynaptic domains was confirmed by the clear co-localization of the transporter protein with the postsynaptic density protein 95 (PSD95). Interestingly, the co-localization of VNUT and the lysosomal marker LAMP-1 in certain cytosolic areas indicated that VNUT can be also found in lysosomes (Menendez-Mendez et al., 2017).

The glutamatergic phenotype of cerebellar granule neurons requires the vesicular storage of glutamate through VGLUTs, of which the most abundant isoform is VGLUT1. Immunofluoresecence assays showed a weak co-localization between VNUT and VGLUT1 in the granule neurons, suggesting that ATP- and glutamate-containing vesicle pools are segregated in these cells (Menendez-Mendez et al., 2017). Nevertheless, it should be taken into account that these studies were performed *in vitro*, in cultured granule cells. To assess whether such distribution also reflects the situation *in vivo*, slices of mouse cerebellum were immunolabeled with antibodies to VNUT and VLGUT1. These two vesicular transporters clearly showed a non-overlapping distribution with only a few examples of co-localization between VGLUT and VNUT in both the molecular or granular layers, which was consistent with the results obtained in cultured cells (Menendez-Mendez et al., 2017).

Cerebellar sections of P5 and P15 mice were used to analyze the distribution of VNUT during the postnatal development of the cerebellar cortex. At P5 stage, cortical layers of the cerebellum are not well defined and immature granule neurons are still migrating from the external to the internal granular layer, where they reach their final location. At this stage, VNUT was abundantly expressed and can be found in granule cell precursors and immature neurons that have not completely differentiated (Menendez-Mendez et al., 2017), suggesting a possible role of this transporter in the initial stages of granule cells maturation. Committed granule cells become mature neurons during the subsequent development of the cerebellum. Consistent with this, markers of neuronal progenitors and immature neurons are dramatically reduced at P15 stage. Nevertheless, VNUT expression persisted at this stage (Menendez-Mendez et al., 2017), indicating the relevance of VNUT during the commitment and differentiation of granule cells. These results correlate with the observations *in vitro*. VNUT expression could be detected from the first day of culture, when cerebellar granule neurons are still immature, persisting once the cells have matured and established synaptic contacts (Menendez-Mendez et al., 2017). Although further studies are required to fully understand the role of VNUT in the maturation of the granule cells and the development of the cerebellum, these findings, showing the localization and activity of VNUT in cerebellar granule neurons *in vitro* and their correlation with the situation *in vivo*, opens exciting new questions that need to be addressed in the future.

**FIGURE 4:**
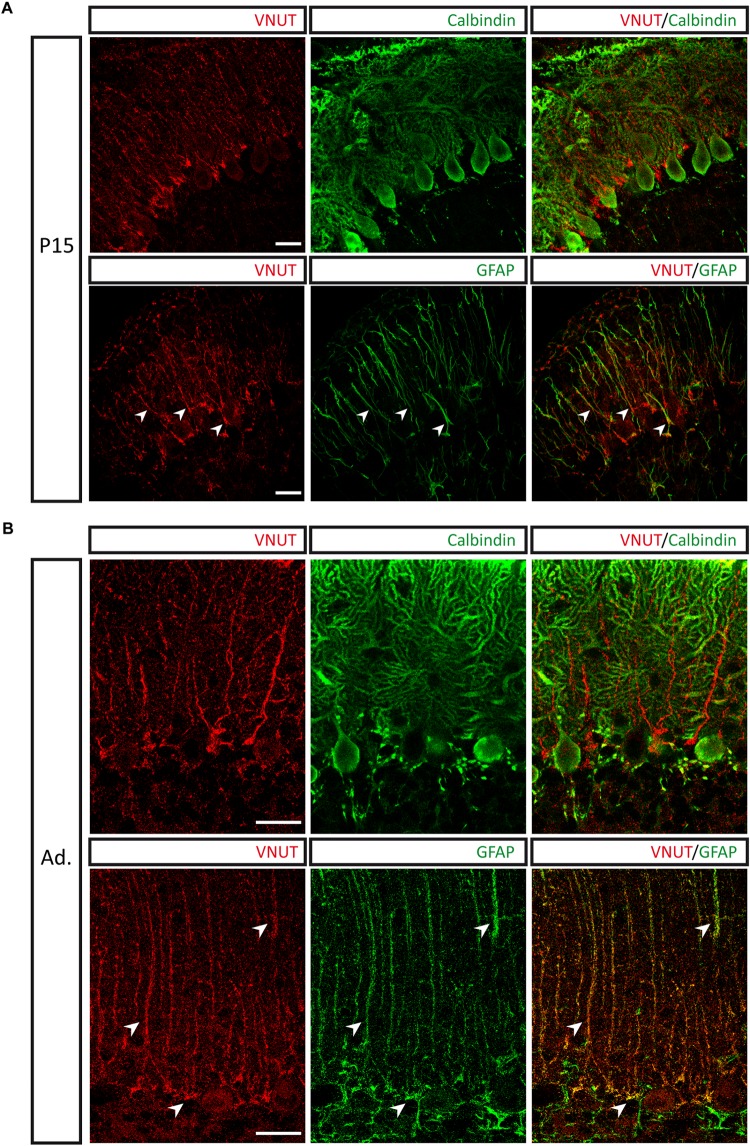
Presence of VNUT in Bergmann glia. Representative immunofluorescence images showing double immunostaining for VNUT (red) and calbindin or GFAP (green) in cerebellar sections of P15 **(A)** and adult **(B)** mice. Arrowheads indicate co-localization of the immunoreactivity for VNUT and GFAP. Scale bar: 20 μm. Adapted from Menéndez Méndez (2017). Images are reproduced with permission of the copyright holders.

Double immunolabeling with antibodies against calbindin and VNUT revealed that the vesicular nucleotide transported is located adjacent to Purkinje neurons in P15 cerebellar sections, showing a filamentous morphology. Besides, co-localization between VNUT and the glial fibrillary acidic protein (GFAP) could be observed (Figure 4A). This staining pattern is consistent with the presence of VNUT in Bergmann glia, a population of radial glia present in the Purkinje cell layer. Moreover, the same pattern of VNUT immunolabeling persisted in the adult stage (Figure 4B), thus indicating that VNUT is present in this type of glial cells both in the advanced stages of cerebellar development and in the adult cerebellum. Bergmann glia has been postulated as one of the neurogenic populations in the cerebellum, as expression of neural stem cell markers, such as Sox2, a transcription factor that plays a key role in neurogenesis during the development of the nervous system, has been described in these cells (Sottile et al., 2006; Alcock et al., 2009). Presence of VNUT in these putative stem cells also supports a potential role of this vesicular transporter in the development of the cerebellum.

#### VNUT in Midbrain Dopaminergic Neurons

Immunohistochemical studies showed the presence of VNUT in tyrosine hydroxylase (TH)-positive dopaminergic neurons of the *substantia nigra* and ventral tegmental area of the midbrain (Ho et al., 2015). All TH-positive dopaminergic neurons in these areas were VNUT-positive. Nevertheless, expression of VNUT was not restricted to dopaminergic neurons, as VNUT-positive TH-negative cells can be detected in the *substantia nigra* and other regions of the brain (Ho et al., 2015). These findings, together with the fact that VNUT is expressed by retinal dopaminergic neurons, which incorporate ATP into vesicles and release ATP when stimulated (see below), indicate that the machinery necessary for vesicular ATP release is present in dopaminergic neurons from different regions of the central nervous system, and that these dopaminergic neurons can be a source of extracellular ATP and its bioactive breakdown products (Ho et al., 2015). These purinergic ligands would mediate their actions trough purinoceptors which are pre-synaptically expressed on dopaminergic neurons and co-expressed with dopamine receptors on neurons in regions that receive the dopaminergic input (Amadio et al., 2007; Morin and Di Paolo, 2014). As dopaminergic neurons in the midbrain are involved in the modulation of a wide range of behaviors, such as motor control, motivation and reward responses (Wise, 2008; Joshua et al., 2009; Cachope and Cheer, 2014), it is likely that purinergic transmission could play a role in these processes. Likewise, purinergic neurotransmission could have a role in neurological disorders involving dysregulation of midbrain dopaminergic pathways, such as Parkinson’s disease (Chinta and Andersen, 2005; Hracsko et al., 2011).

### VNUT in Glial Cells

#### VNUT in Astrocytes

Several studies have shown that astrocytes respond to neurotransmitters and release different gliotransmitters, including ATP, which can trigger propagation of Ca^2+^ waves in these cells and modulate the activity of surrounding neurons (Stout et al., 2002; Coco et al., 2003; Newman, 2003; Zhang et al., 2003; Bowser and Khakh, 2007; Chen et al., 2013). However, the precise mechanism by which astrocytes release ATP are not well understood and both Ca^2+^-dependent exocytosis (Coco et al., 2003; Pascual et al., 2005; Pangrsic et al., 2007; Parpura and Zorec, 2010; Lalo et al., 2014) and non-vesicular release pathways (Stout et al., 2002; Suadicani et al., 2006, 2012; Liu et al., 2008) have been described. Lysosomes can be a relevant source of vesicular ATP release from astrocytes, with the fusion of lysosomal and plasma membranes leading to ATP exocytosis (Zhang et al., 2007; Verderio et al., 2012).

Vesicular nucleotide transporter is expressed in C6 glioma cells and primary cultures of cortical astrocytes, where it is mainly associated to lysosomes, as demonstrated by the co-localization of VNUT-EGFP with lysosomal markers (Oya et al., 2013). Interestingly, VNUT-associated lysosomes release their content in response to elevations of the intracellular Ca^2+^ concentration, but they do not completely collapse into the plasma membrane after lysosomal exocytosis, as VNUT remains associated with the secretory lysosome and failed to spread into the plasma membrane, suggesting that lysosomes retain their spherical structure for a long time after fusion to plasma membrane. Such “kiss and stay” mechanism is quite different from the behavior of synaptic or dense-core vesicles, which easily spread into the plasma membrane during fusion events (Oya et al., 2013). ATP uptake into the secretory lysosomes decreased by pharmacological inhibition of VNUT by Evans blue. Moreover, silencing of VNUT expression by siRNA or inhibition of VNUT function by Evans blue reduced the amount of ATP released by the cells, whereas overexpression of VNUT increased it. Collectively, these data demonstrate the implication of VNUT in ATP storage in secretory lysosomes in astrocytes and its relevant role in astrocytic ATP release by lysosomal exocytosis (Oya et al., 2013). VNUT co-localize with the lysosomal marker LAMP3 in rat optic nerve head astrocytes that release lysosomal ATP after stimulation of the toll-like receptor 3 (TLR3), which is an additional evidence supporting the role of VNUT in the storage of ATP in secretory lysosomes in astrocytes (Beckel et al., 2018).

Astrocytes residing near the brainstem ventral surface (central respiratory chemosensitive area) respond to physiological reductions in pH with elevations in intracellular Ca^2+^ and ATP release. ATP stimulates the brainstem respiratory network, thus contributing to adaptive changes in lung ventilation. In terms of their sensitivity to pH, ventral brainstem astrocytes clearly differ from astrocytes that reside in other parts of the brain, such as cerebral cortex astrocytes, which indicates that these cells are functionally specialized (Kasymov et al., 2013). Compared to cortical astrocytes, ventral brainstem astrocytes showed increased levels of expression of genes encoding proteins associated with vesicular ATP transport and vesicular fusion, including VNUT (Kasymov et al., 2013), which suggests that astrocytes of the brainstem chemosensitive area are able to respond to acidification with enhanced vesicular release of ATP. Moreover, ATP released from astrocytes, possibly by the exocytosis of VNUT-possesing vesicular compartments, is involved in sensing of physiological changes in oxygen concentration in the brain (Angelova et al., 2015).

ATP derived from astrocytes modulates depressive behaviors in mice (Cao et al., 2013). In this sense, VNUT-dependent ATP release from astrocytes seems to play a pivotal role in the therapeutic effect of the anti-depressant fluoxetine (Kinoshita et al., 2018). Fluoxetine increases exocytotic ATP release in primary cultures of hippocampal astrocytes. Fluoxetine-induced ATP release was significantly reduced in astrocytes obtained from VNUT-knockdown mice, indicating that fluoxetine, at least in part, stimulates the release of ATP by VNUT-dependent exocytosis (Kinoshita et al., 2018). Fluoxetine-induced anti-depressive behavior was decreased in VNUT-knockdown mice and, relevantly, the anti-depressive effects of fluoxetine were dependent on astrocytic VNUT, as demonstrated in mice with selective knockout or overexpression of the VNUT gene in astrocytes. A decrease or increase of VNUT in astrocytes, resulted in a decrease or increase in the anti-depressive effects induced by fluoxetine, respectively. These findings demonstrate that fluoxetine acts on astrocytes and mediates its anti-depressive effect by increasing VNUT-dependent ATP exocytosis from these cells (Kinoshita et al., 2018). Released ATP and its metabolite adenosine act on P2Y_11_ and adenosine A2b receptors expressed by astrocytes, inducing an increase in brain-derived neurotrophic factor (BDNF), which is considered to have a relevant role in the therapeutic action of anti-depressants (Kinoshita et al., 2018).

#### VNUT in the Microglia

Microglial cells constitute the resident immune cell population of the mammalian central nervous system. These cells monitor environmental changes and act as damage sensors within the brain. Any type of injury or pathological process induces the activation of the microglia, which change their morphology, migrate to the site of injury, proliferate, produce/release several substances that affect pathological processes, or even phagocytose damaged cells or debris to restore the brain homeostasis (Kettenmann et al., 2011). Extracellular nucleotides are relevant mediators that regulate the function of the microglia and, consequently, purinoceptor-mediated microglial responses have received much attention (Calovi et al., 2018). Microglia release ATP in response to different stimuli, such as ATP or glutamate (Liu et al., 2006; Dou et al., 2012). The mechanisms involved in ATP release from microglia remain unclear, although participation of connexin 43 (Cx43) hemichannels has been described (Ma et al., 2014).

Vesicular nucleotide transporter was found to be expressed in vesicular-like structures in primary cultured microglia and exhibited no co-localization with lysosomes (Imura et al., 2013). When cells where incubated with the fluorescent ATP analog 2′/3′-O-(*N*-Methyl-anthraniloyl)-adenosine-5′-triphosphate (MANT-ATP), the existence of many ATP-possessing vesicular structures could be observed. Microglia release ATP in an exocytotic manner: when cells were stimulated with ionomycin they released ATP and such release was dependent on Ca^2+^, vesicular H^+^-ATPase and soluble *N*-ethylmaleimide sensitive factor attachment protein receptors (SNAREs), but independent on connexin/pannexin hemichannels. Additionally, exocytotic events of ATP-containing vesicles could be visualized by quinacrine-based TIRF microscopy in the microglial cell line MG5 (Imura et al., 2013). Ionomycin-induced ATP release from microglial cells was dependent upon VNUT, as release was significantly reduced when cells were treated with VNUT-siRNA. These findings demonstrated that microglia possess functional VNUT by which microglial cells store and release ATP in an exocytotic process (Imura et al., 2013). Moreover, stimulation of MG5 cells with the bacterial endotoxin lipopolysaccharide (LPS) significantly increased ionomycin-evoked ATP release, which was associated with an increase in VNUT expression. The increase in ATP release by LPS was abolished by the knockdown of VNUT (treatment of the cells with VNUT siRNA), indicating that the increase in VNUT by LPS should be responsible for the enhancement of the ATP release in microglial MG5 cells. This could be of relevance because, during infections or brain injuries, microglia could increase exocytotic ATP release by increasing VNUT (Imura et al., 2013).

Methylmercury (MeHg) is a well-known environmental pollutant that easily crosses the blood-brain barrier, inducing severe neuronal damage. It has been described that cultured microglia sense low concentrations of MeHg and release ATP in response to this neurotoxicant (Shinozaki et al., 2014). MeHg-evoked ATP release is significantly reduced by treatment with the botulinum toxin A, a toxin that cleaves synaptosomal-associated proteins (SNAPs), thereby preventing exocytosis. Moreover, microglia cultures prepared from VNUT knockout mice showed no ATP release when exposed to MeHg, in contrast to the significant ATP release from wild-type microglia. These results indicate that MeHg stimulates the exocytic release of ATP from microglial cells via a VNUT-dependent pathway (Shinozaki et al., 2014). The microglia-derived ATP in turn stimulates P2Y_1_ receptors in astrocytes, which induces the release of interleukin-6 (IL-6), thus protecting neurons against MeHg. These neuroprotective effects were observed in organotypic slices from the hippocampus of wild-type mice, but not in slices obtained from VNUT knockdown mice, where MeHg failed to induce ATP release or IL-6production, which resulted in neuronal damage induced by MeHg (Shinozaki et al., 2014).

## Role of VNUT in Central Nervous System Physiology and Disease

### Role of VNUT in the Regulation of Neuronal Differentiation and Neuritogenesis

Differentiation of the axon is a pivotal process that gives rise to a complex morphology and physiology of neurons. Axon formation and growth is regulated by a variety of extracellular mediators, such as neurotransmitters, neurotrophic factors and other signaling molecules. Stimulating cultured hippocampal neurons with ATP evokes Ca^2+^ transients in the distal part of the axon which exerts a negative effect on axon growth, reducing both axonal length and branching (Diaz-Hernandez et al., 2008). This effect is mediated through P2X7 receptors that are expressed at the growth cone of the axon. Either the pharmacological inhibition of P2X7 receptor or its silencing by shRNA results in longer and more-branched axons, which is coupled to morphological changes of the growth cone (Diaz-Hernandez et al., 2008). This effect of ATP on axonal growth was corroborated by the finding that tissue-nonspecific alkaline phosphatase (TNAP) regulates axonal elongation and branching in hippocampal neurons by controlling the local availability of growth-inhibiting extracellular ATP (Diez-Zaera et al., 2011). Moreover, P2X7 receptors negatively regulate neurite formation in mouse Neuro-2a (N2a) neuroblastoma cell line (Gomez-Villafuertes et al., 2009). Pharmacological inhibition and interference of P2X7 receptor expression were associated with neuritogenesis in N2a cells, whereas P2X7 overexpression significantly reduced neurite formation. Neurotrophic effects of P2X7 were mediated through the modulation of the activity of the Ca^2+^/calmodulin-dependent kinase II and some of its downstream effectors, which have been related to axonal growth and neuronal differentiation processes (Gomez-Villafuertes et al., 2009). Thus, N2a cells have been shown to be a suitable model to analyze the sequence of purinergic events that regulate neuronal differentiation.

Although N2a cells express very small amounts of endogenous VNUT, this transporter can be successfully heterologously expressed in this cell line, and co-localization of VNUT with the vesicular marker synaptophysin could be observed by confocal microscopy imaging (Menendez-Mendez et al., 2015). Retinoic acid-induced differentiation keeps VNUT expression in transfected N2a cells. Functionality of the vesicular transporter was assessed by luciferin-luciferase assays to measure ionomycin-induced ATP release from differentiated N2a cells (Menendez-Mendez et al., 2015). Expression of VNUT clearly decreases neuritogenesis in retinoic acid-treated N2a cells, as both the number and length of neurites are reduced when compared to control (VNUT non-expressing) cells. To corroborate the VNUT negative effect on neuritogenesis, shVNUT was used to knockdown VNUT expression in VNUT-transfected and differentiated cells. These cells, where VNUT expression was reduced, showed more prominent neuritogenesis, with an increase in both the number and length of neurites. These results highlight the role of VNUT as a key component in the sequence of events involved in extracellular ATP regulation of neuritogenesis and neuronal differentiation processes (Menendez-Mendez et al., 2015).

### VNUT in the Spinal Cord: Role of VNUT-Dependent ATP Release in Neuropathic Pain

In the spinal cord, VNUT has been related to neuropathic pain, a hypersensitivity to pain that occurs after damage of a peripheral nerve as a consequence of traumatic injury or diseases such as diabetes mellitus, multiple sclerosis or cancer. Accumulating evidence indicated the crucial role of microglial cells in the spinal cord in the development of neuropathic pain. After damage of a peripheral nerve, spinal microglia turn into a reactive state through a sequence of molecular and cellular changes, which include the increase in the expression of genes that encode purinergic receptors, such as P2X4 or P2Y_12_. In response to the activation of these ATP receptors, microglial cells release different bioactive factors that cause abnormal neurotransmission in the nociceptive network in spinal dorsal horn (SDH). These pathological alterations result in pain hypersensitivity that converts initially innocuous stimuli into nociceptive signals (Tsuda et al., 2003; Tozaki-Saitoh et al., 2008).

Spinal dorsal horn neurons have been identified as a source of extracellular ATP that contributes to peripheral nerve injury (PNI) induced pain hypersensitivity (Masuda et al., 2016). PNI increases VNUT expression in the spinal cord and this upregulation of VNUT is required for the development of tactile allodynia (abnormal pain hypersensitivity evoked by innocuous stimuli), as the intrathecal administration of siRNA targeting *VNUT* in mice subjected to PNI, significantly reduced the expression of spinal VNUT and ameliorated PNI-evoked allodynia. Moreover, PNI also increased extracellular ATP content within the spinal cord and this increase was suppressed by vesicular exocytosis inhibitors (Masuda et al., 2016). VNUT-deficient (*VNUT^-/-^*) mice did not shown PNI-evoked increase in extracellular ATP concentration and had attenuated pain hypersensitivity. Attenuation of PNI-induced allodynia and reduction in spinal extracellular ATP content was also observed in mice with specific deletion of VNUT in SDH neurons, but not in mice lacking VNUT in primary sensory neurons, astrocytes or microglia. Moreover, ectopic expression of VNUT in SDH neurons of VNUT-deficient mice restored PNI-evoked increase in extracellular ATP and pain. These results showed that VNUT-dependent exocytotic ATP release from dorsal horn neurons is an essential mechanism for neuropathic pain after PNI (Masuda et al., 2016).

Vesicular nucleotide transporter is expressed in subpopulations of rat dorsal root ganglion (DRG) neurons (Nishida et al., 2014). In a model of rats subjected to nerve injury (L5 spinal nerve ligation), an increase in VNUT expression was observed in injured DRG neurons. Moreover, VNUT co-localize with the lysosomal protein LAMP1 in these cells (Jung et al., 2016). Fluorescent labeling of lysosomal vesicles demonstrated that ATP containing VNUT-positive lysosomes are transported to the central nerve terminals of DRG neurons in the dorsal horn. Although there is no direct evidence of ATP release from primary afferent nerve terminals in dorsal horn through lysosomal exocytosis, a previous *in vitro* study revealed exocytotic ATP release from lysosomes in primary cultured DRG neurons (Jung et al., 2013). In the light of these findings, it has been suggested that lysosomal exocytosis from central terminals of DRG neurons could be an additional source of ATP that contribute to activation of the microglia in the dorsal horn after nerve injury (Jung et al., 2016).

Vesicular nucleotide transporter shows strong similarities with other members of the family of SCL17 anion transporters, such as the vesicular glutamate transporter (VGLUT). VGLUT undergoes unusual regulation by Cl^-^. Glutamate uptake into vesicles shows biphasic dependence with the concentration of this ion: low Cl^-^ concentrations (2–8 mM) stimulate glutamate transport while high concentrations (>20 mM) inhibit it. It has been suggested that Cl^-^ acts as an allosteric modulator of VGLUT that triggers glutamate uptake upon binding to the transporter (Juge et al., 2010), whereas the inhibition of vesicular glutamate accumulation by high Cl^-^ concentrations could be related to the dissipation of Δψ, the component of ΔμH^+^ that drives vesicular glutamate uptake. However, the precise molecular mechanism underlying the regulation of VGLUT by Cl^-^ is still to be clarified. Ketone bodies inhibit vesicular glutamate transport by competing with Cl^-^ at the site of allosteric regulation (Juge et al., 2010), which suggest a metabolic control of vesicular glutamate release. The strong dependence on transport activity with Cl^-^ concentration is a characteristic shared by other members of the SLC17 family such as VNUT. Presence of this ion is an absolute requirement for ATP transport activity in VNUT, as nucleotide transport cannot be detected in the absence of Cl^-^. Br^-^ also activates transport, whereas I^-^, F^-^, nitrate, sulfate, and thiocyanate are ineffective (Sawada et al., 2008). This anion dependence is very similar to that of VGLUT2 (Juge et al., 2010). Moreover, VNUT is also inhibited by ketone bodies such as acetoacetate (Figure 1B), the inhibitory effect being reversible and prevented by high concentrations of Cl^-^ (Juge et al., 2010). Although detailed kinetic studies have not been carried out, these findings suggest the existence of similar anion binding sites and regulatory mechanisms in both members of the SLC17 family of transporters, VNUT and VGLUT.

Clodronate is a first-generation bisphosphonate used in antiresorptive therapy for osteoporosis. Nevertheless, studies have proved that clodronate also has analgesic properties, although the mechanism underlying this analgesic effect was unknown. Recently, clodronate was identified as a potent and selective inhibitor of ATP vesicular storage and release (Figure 1B; Kato et al., 2017). *In vitro* assays demonstrated that clodronate inhibits VNUT (IC_50_ = 15.6 nM) without affecting other vesicular neurotransmitter transporters, acting as an allosteric modulator that interacts with the Cl^-^ binding site. Clodronate shifted the Cl^-^ concentration necessary for VNUT activation toward a higher activation level, suggesting a competitive interaction. Consistent with this, clodronate modulates vesicular ATP release. Low concentrations of clodronate completely inhibited ATP release from microglia, neurons and immune cells (human monocyte cell line THP-1). *In vivo* analysis revealed that clodronate attenuates neuropathic and inflammatory pain, in addition to the accompanying inflammation, in wild type but no *VNUT^-/-^* mice, without altering basal nociception (Kato et al., 2017). These results demonstrated that clodronate exerts analgesic and anti-inflammatory actions by targeting VNUT. Noticeably, clodronate is approved for clinical use in the treatment of osteoporosis and its clinical safety in humans is well established (Muratore et al., 2011). Moreover, clodronate attenuates inflammatory and neuropathic pain with stronger, faster acting, and longer lasting effects than existing drugs (Kato et al., 2017; Moriyama and Nomura, 2018). Thereby, clodronate is likely to be clinically useful in the treatment of chronic neuropathic pain and could open new perspectives regarding the use of VNUT inhibitors that block vesicular ATP release as therapeutic drugs.

### VNUT in the Retina: Possible Role of VNUT in the Development of Glaucoma

Using laser microdissected retinal samples, VNUT mRNA expression was detected in photoreceptor and inner nuclear layer/ganglion cell layer (INL/GCL) samples (Vessey and Fletcher, 2012). Immunochemical studies have shown that VNUT appear to be widely distributed throughout the inner and outer retinal layers, with particular strong immunoreactivity detected in the outer segments of photoreceptors, outer plexiform layer, inner plexiform layer and ganglion cell layer. Presence of VNUT in these retinal areas was confirmed by the loss of VNUT immunoreactivity in the retina from VNUT knockout (*VNUT^-/-^*) mice (Moriyama and Hiasa, 2016). Double-labeling immunochemistry showed that VNUT is co-localized with synaptophysin and VGLUT1 in photoreceptor cells, whereas it is co-localized with vesicular γ-aminobutyric acid (GABA) transporter (VGAT) in bipolar and amacrine cells. VNUT is also present in astrocytes and Müller cells. Retinal membrane fraction took up radiolabeled ATP in a DIDS and bafilomycin A1 (a vacuolar ATPase inhibitor) sensitive manner, this ATP-uptake activity being absent in retinal membrane vesicles prepared from *VNUT^-/-^* mice (Moriyama and Hiasa, 2016). Thus, these results indicate that VNUT is widely present in retina, where ATP can be stored and released to initiate purinergic chemical transmission.

Vesicular nucleotide transporter immunoreactivity can be detected in TH positive dopaminergic amacrine/interplexiform cells (Ho et al., 2015). Three-dimensional reconstruction of retinal flatmounts immunolabelled with VNUT showed that VNUT-positive amacrine/plexiform cells processes are closely associated with cone photoreceptors terminals and horizontal cells, which are known to express P2 purinergic receptors. In order to assess function, dissociated retinal neurons were loaded with fluorescent dopamine (FFN102) and ATP (MANT-ATP, quinacrine) markers and immunostained with a VNUT antibody. VNUT-immunoreactive neurons load fluorescent ATP and dopamine markers in vesicles. Moreover, ATP and dopamine markers co-localize in these cells, thus indicating co-loading of ATP and dopamine in vesicles within the VNUT-positive neurons. Fluorescence of the ATP marker quinacrine was reduced upon K^+^ stimulation, this response being blocked in the presence of cadmium. Taken together, all these results indicate that dopaminergic neurons in the retina release ATP via calcium dependent exocytosis, which may modulate the visual response by stimulating purinergic receptors in closely associated cells (Ho et al., 2015).

Retinal extracellular ATP levels and changes in VNUT expression have been analyzed in the DBA/2J mouse model of glaucoma during the development of the disease (Perez de Lara et al., 2015). For this purpose, retinas were dissected from glaucomatous animals at 3, 9, 15, and 22 months of age. C57BL/6J mice were used as age-matched controls. Retinal net ATP release increased with the progression of the pathology, varying from 0.32 pmol/retina (3 months) to 1.10 pmol/retina (15 months, threefold increase). Concomitantly, a significant increase in VNUT expression in DBA/2J mice retina during glaucoma progression was detected. These data may suggest a possible correlation between retinal dysfunction and increased levels of extracellular ATP and nucleotide transporter (Perez de Lara et al., 2015).

## Concluding Remarks

It is widely accepted that VNUT is responsible for the storage of ATP and other nucleotides into secretory vesicles and therefore plays an essential role in the vesicular release of nucleotides and the initiation of purinergic chemical transmission. Both the reduction in the expression of VNUT and the inhibition of its activity reduce the vesicular release of ATP and lead to a decrease in purinergic chemical signaling. VNUT appear to be widely expressed in the central nervous system, being present in neurons, astrocytes and microglial cells. Accumulating evidence indicate the involvement of VNUT-dependent nucleotide release in a diversity of biological processes in the central nervous system, which include development of the cerebellar cortex, neuronal differentiation and neuritogenesis, sensing of physiological changes in pH and brain oxygenation, protection against neurotoxicants or modulation of depressive behaviors. The expression pattern, localization and functions of VNUT in the central nervous system are summarized in Table 1. Deficiencies in the vesicular release of ATP could have beneficial effects in certain pathological conditions. In particular, increased levels of extracellular ATP have correlated with retinal dysfunction during the development of glaucoma. Moreover, mice deficient in VNUT show attenuated neuropathic pain, and the selective inhibitor of VNUT, clodronate, exerts analgesic effects. Therefore, VNUT could constitute a new and relevant molecular target in the context of the pathophysiology of purinergic transmission. Impairment of purinergic signaling by inhibiting the activity of VNUT or silencing VNUT gene expression may represent a new and promising therapeutical strategy for the treatment of a variety of pathological conditions.

**Table 1:** Expression, localization, and function of VNUT in the central nervous system.

Organ	Location (vesicle)	Role	References
Brain	Hippocampal neurons (synaptic vesicles, postsynaptic vesicular structures)	VNUT-dependent ATP release	Larsson et al., 2012; Sakamoto et al., 2014
	Cerebellar granule neurons (synaptic vesicles, lysosomes)	Granule cell development	Menendez-Mendez et al., 2017
	Bergmann glia	ND	Menéndez Méndez, 2017
	Midbrain dopaminergic neurons	Vesicular ATP release	Ho et al., 2015
	Cortical astrocytes (lysosomes)	Lysosomal ATP release	Oya et al., 2013; Beckel et al., 2018
	Brainstem astrocytes	Response to changes in pH and brain oxygenation	Kasymov et al., 2013; Angelova et al., 2015
	Hippocampal astrocytes	Effect of antidepressants	Kinoshita et al., 2018
	Microglia (vesicular-like structures)	Exocytotic ATP release. Neuroprotective response to neurotoxicants	Imura et al., 2013; Shinozaki et al., 2014
Spinal cord	Dorsal horn neurons	Neuropathic pain	Masuda et al., 2016
	Dorsal root ganglion neurons (lysosomes)	Microglial activation in dorsal horn after nerve injury	Nishida et al., 2014; Jung et al., 2016
Retina	Photoreceptor cells, bipolar cells, astrocytes, Müller cells	ND	Moriyama and Hiasa, 2016
	Amacrine cells	Calcium-dependent ATP exocytosis	Ho et al., 2015; Moriyama and Hiasa, 2016


*ND, not determined.*

## Author Contributions

JG wrote the manuscript and made illustrations. MM-P, AM-M, RG-V, FO, ED, and RP-S wrote the manuscript.

## Conflict of Interest Statement

The authors declare that the research was conducted in the absence of any commercial or financial relationships that could be construed as a potential conflict of interest.

## Footnotes

1 http://portal.brain-map.org/

## References

[B1] Abbracchio M. P. Burnstock G. Verkhratsky A. Zimmermann H. (2009). Purinergic signalling in the nervous system: an overview. *Trends Neurosci.* 32 19–29. 10.1016/j.tins.2008.10.001 19008000

[B2] Aberer W. Kostron H. Huber E. Winkler H. (1978). A characterization of the nucleotide uptake of chromaffin granules of bovine adrenal medulla. *Biochem. J.* 172 353–360. 10.1042/bj1720353b 28725 PMC1185708

[B3] Alcock J. Lowe J. England T. Bath P. Sottile V. (2009). Expression of Sox1, Sox2 and Sox9 is maintained in adult human cerebellar cortex. *Neurosci. Lett.* 450 114–116. 10.1016/j.neulet.2008.11.047 19061938

[B4] Amadio S. Montilli C. Picconi B. Calabresi P. Volonte C. (2007). Mapping P2X and P2Y receptor proteins in striatum and substantia nigra: an immunohistological study. *Purinergic Signal.* 3 389–398. 10.1007/s11302-007-9069-8 18404452 PMC2072921

[B5] Angelova P. R. Kasymov V. Christie I. Sheikhbahaei S. Turovsky E. Marina N. (2015). Functional oxygen sensitivity of astrocytes. *J. Neurosci.* 35 10460–10473. 10.1523/JNEUROSCI.0045-15.2015 26203141 PMC4510287

[B6] Bankston L. A. Guidotti G. (1996). Characterization of ATP transport into chromaffin granule ghosts. synergy of ATP and serotonin accumulation in chromaffin granule ghosts. *J. Biol. Chem.* 271 17132–17138. 10.1074/jbc.271.29.17132 8663306

[B7] Baroja-Mazo A. Barbera-Cremades M. Pelegrin P. (2013). The participation of plasma membrane hemichannels to purinergic signaling. *Biochim. Biophys. Acta* 1828 79–93. 10.1016/j.bbamem.2012.01.002 22266266

[B8] Beckel J. M. Gomez N. M. Lu W. Campagno K. E. Nabet B. Albalawi F. (2018). Stimulation of TLR3 triggers release of lysosomal ATP in astrocytes and epithelial cells that requires TRPML1 channels. *Sci. Rep.* 8:5726. 10.1038/s41598-018-23877-3 29636491 PMC5893592

[B9] Blakely R. D. Edwards R. H. (2012). Vesicular and plasma membrane transporters for neurotransmitters. *Cold Spring Harb. Perspect. Biol.* 4:a005595. 10.1101/cshperspect.a005595 22199021 PMC3281572

[B10] Bowser D. N. Khakh B. S. (2007). Vesicular ATP is the predominant cause of intercellular calcium waves in astrocytes. *J. Gen. Physiol.* 129 485–491. 10.1085/jgp.200709780 17504911 PMC2151627

[B11] Burnstock G. (2007a). Physiology and pathophysiology of purinergic neurotransmission. *Physiol. Rev.* 87 659–797. 10.1152/physrev.00043.2006 17429044

[B12] Burnstock G. (2007b). Purine and pyrimidine receptors. *Cell. Mol. Life Sci.* 64 1471–1483. 17375261 10.1007/s00018-007-6497-0 PMC11149472

[B13] Burnstock G. Krugel U. Abbracchio M. P. Illes P. (2011). Purinergic signalling: from normal behaviour to pathological brain function. *Prog. Neurobiol.* 95 229–274. 10.1016/j.pneurobio.2011.08.006 21907261

[B14] Cachope R. Cheer J. F. (2014). Local control of striatal dopamine release. *Front. Behav. Neurosci.* 8:188. 10.3389/fnbeh.2014.00188 24904339 PMC4033078

[B15] Calovi S. Mut-Arbona P. Sperlagh B. (2018). Microglia and the purinergic signaling system. *Neuroscience* 405 137–147. 10.1016/j.neuroscience.2018.12.021 30582977

[B16] Cao X. Li L. P. Wang Q. Wu Q. Hu H. H. Zhang M. (2013). Astrocyte-derived ATP modulates depressive-like behaviors. *Nat. Med.* 19 773–777. 10.1038/nm.3162 23644515

[B17] Chaudhry F. A. Edwards R. H. Fonnum F. (2008). Vesicular neurotransmitter transporters as targets for endogenous and exogenous toxic substances. *Annu. Rev. Pharmacol. Toxicol.* 48 277–301. 10.1146/annurev.pharmtox.46.120604.141146 17883368

[B18] Chen J. Tan Z. Zeng L. Zhang X. He Y. Gao W. (2013). Heterosynaptic long-term depression mediated by ATP released from astrocytes. *Glia* 61 178–191. 10.1002/glia.22425 23044720

[B19] Chinta S. J. Andersen J. K. (2005). Dopaminergic neurons. *Int. J. Biochem. Cell Biol.* 37 942–946. 10.1016/j.biocel.2004.09.009 15743669

[B20] Coco S. Calegari F. Pravettoni E. Pozzi D. Taverna E. Rosa P. (2003). Storage and release of ATP from astrocytes in culture. *J. Biol. Chem.* 278 1354–1362. 10.1074/jbc.M209454200 12414798

[B21] Diaz-Hernandez M. del Puerto A. Diaz-Hernandez J. I. Diez-Zaera M. Lucas J. J. Garrido J. J. (2008). Inhibition of the ATP-gated P2X7 receptor promotes axonal growth and branching in cultured hippocampal neurons. *J. Cell. Sci.* 121(Pt 22), 3717–3728. 10.1242/jcs.034082 18987356

[B22] Diez-Zaera M. Diaz-Hernandez J. I. Hernandez-Alvarez E. Zimmermann H. Diaz-Hernandez M. Miras-Portugal M. T. (2011). Tissue-nonspecific alkaline phosphatase promotes axonal growth of hippocampal neurons. *Mol. Biol. Cell.* 22 1014–1024. 10.1091/mbc.E10-09-0740 21289095 PMC3069005

[B23] Dou Y. Wu H. J. Li H. Q. Qin S. Wang Y. E. Li J. (2012). Microglial migration mediated by ATP-induced ATP release from lysosomes. *Cell Res.* 22 1022–1033. 10.1038/cr.2012.10 22231629 PMC3367529

[B24] Eiden L. E. Schafer M. K. Weihe E. Schutz B. (2004). The vesicular amine transporter family (SLC18): amine/proton antiporters required for vesicular accumulation and regulated exocytotic secretion of monoamines and acetylcholine. *Pflugers Arch.* 447 636–640. 10.1007/s00424-003-1100-5 12827358

[B25] Gasnier B. (2004). The SLC32 transporter, a key protein for the synaptic release of inhibitory amino acids. *Pflugers Arch.* 447 756–759. 10.1007/s00424-003-1091-2 12750892

[B26] Gomez-Villafuertes R. del Puerto A. Diaz-Hernandez M. Bustillo D. Diaz-Hernandez J. I. Huerta P. G. (2009). Ca2+/calmodulin-dependent kinase II signalling cascade mediates P2X7 receptor-dependent inhibition of neuritogenesis in neuroblastoma cells. *FEBS J.* 276 5307–5325. 10.1111/j.1742-4658.2009.07228.x 19682070

[B27] Gualix J. Abal M. Pintor J. Garcia-Carmona F. Miras-Portugal M. T. (1996). Nucleotide vesicular transporter of bovine chromaffin granules. Evidence for a mnemonic regulation. *J. Biol. Chem.* 271 1957–1965. 10.1074/jbc.271.4.1957 8567644

[B28] Gualix J. Alvarez A. M. Pintor J. Miras-Portugal M. T. (1999a). Studies of chromaffin granule functioning by flow cytometry: transport of fluorescent epsilon-ATP and granular size increase induced by ATP. *Receptors Channels* 6 449–461. 10635062

[B29] Gualix J. Pintor J. Miras-Portugal M. T. (1999b). Characterization of nucleotide transport into rat brain synaptic vesicles. *J. Neurochem.* 73 1098–1104. 10.1046/j.1471-4159.1999.0731098.x 10461900

[B30] Gualix J. Fideu M. D. Pintor J. Rotllan P. Garcia-Carmona F. Miras-Portugal M. T. (1997). Characterization of diadenosine polyphosphate transport into chromaffin granules from adrenal medulla. *FASEB J.* 11 981–990. 10.1096/fasebj.11.12.9337151 9337151

[B31] Gualix J. Gomez-Villafuertes R. Pintor J. Llansola M. Felipo V. Miras-Portugal M. T. (2014). Presence of diadenosine polyphosphates in microdialysis samples from rat cerebellum in vivo: effect of mild hyperammonemia on their receptors. *Purinergic Signal.* 10 349–356. 10.1007/s11302-013-9382-3 23943472 PMC4040178

[B32] Ho T. Jobling A. I. Greferath U. Chuang T. Ramesh A. Fletcher E. L. (2015). Vesicular expression and release of ATP from dopaminergic neurons of the mouse retina and midbrain. *Front. Cell. Neurosci.* 9:389. 10.3389/fncel.2015.00389 26500494 PMC4593860

[B33] Holmsen H. Weiss H. J. (1979). Secretable storage pools in platelets. *Annu. Rev. Med.* 30 119–134. 10.1146/annurev.me.30.020179.001003 233610

[B34] Hracsko Z. Baranyi M. Csolle C. Goloncser F. Madarasz E. Kittel A. (2011). Lack of neuroprotection in the absence of P2X7 receptors in toxin-induced animal models of parkinson’s disease. *Mol. Neurodegener.* 6:28. 10.1186/1750-1326-6-28 21542899 PMC3113297

[B35] Hutton J. C. Penn E. J. Peshavaria M. (1983). Low-molecular-weight constituents of isolated insulin-secretory granules. Bivalent cations, adenine nucleotides and inorganic phosphate. *Biochem. J.* 210 297–305. 10.1042/bj2100297 6344863 PMC1154224

[B36] Imura Y. Morizawa Y. Komatsu R. Shibata K. Shinozaki Y. Kasai H. (2013). Microglia release ATP by exocytosis. *Glia* 61 1320–1330. 10.1002/glia.22517 23832620

[B37] Jo Y. H. Schlichter R. (1999). Synaptic corelease of ATP and GABA in cultured spinal neurons. *Nat. Neurosci.* 2 241–245. 10.1038/6344 10195216

[B38] Johnson R. G. Jr. (1988). Accumulation of biological amines into chromaffin granules: a model for hormone and neurotransmitter transport. *Physiol. Rev.* 68 232–307. 10.1152/physrev.1988.68.1.232 2892215

[B39] Joshua M. Adler A. Bergman H. (2009). The dynamics of dopamine in control of motor behavior. *Curr. Opin. Neurobiol.* 19 615–620. 10.1016/j.conb.2009.10.001 19896833

[B40] Juge N. Gray J. A. Omote H. Miyaji T. Inoue T. Hara C. (2010). Metabolic control of vesicular glutamate transport and release. *Neuron* 68 99–112. 10.1016/j.neuron.2010.09.002 20920794 PMC2978156

[B41] Jung J. Shin Y. H. Konishi H. Lee S. J. Kiyama H. (2013). Possible ATP release through lysosomal exocytosis from primary sensory neurons. *Biochem. Biophys. Res. Commun.* 430 488–493. 10.1016/j.bbrc.2012.12.009 23237805

[B42] Jung J. Uesugi N. Jeong N. Y. Park B. S. Konishi H. Kiyama H. (2016). Increase of transcription factor EB (TFEB) and lysosomes in rat DRG neurons and their transportation to the central nerve terminal in dorsal horn after nerve injury. *Neuroscience* 313 10–22. 10.1016/j.neuroscience.2015.11.028 26601776

[B43] Kasymov V. Larina O. Castaldo C. Marina N. Patrushev M. Kasparov S. (2013). Differential sensitivity of brainstem versus cortical astrocytes to changes in pH reveals functional regional specialization of astroglia. *J. Neurosci.* 33 435–441. 10.1523/JNEUROSCI.2813-12.2013 23303924 PMC3690976

[B44] Kato Y. Hiasa M. Ichikawa R. Hasuzawa N. Kadowaki A. Iwatsuki K. (2017). Identification of a vesicular ATP release inhibitor for the treatment of neuropathic and inflammatory pain. *Proc. Natl. Acad. Sci. U.S.A.* 10.1073/pnas.1704847114 [Epub ahead of print]. 28720702 PMC5547629

[B45] Kettenmann H. Hanisch U. K. Noda M. Verkhratsky A. (2011). Physiology of microglia. *Physiol. Rev.* 91 461–553. 10.1152/physrev.00011.2010 21527731

[B46] Kinoshita M. Hirayama Y. Fujishita K. Shibata K. Shinozaki Y. Shigetomi E. (2018). Anti-Depressant fluoxetine reveals its therapeutic effect via astrocytes. *EBioMedicine* 32 72–83. 10.1016/j.ebiom.2018.05.036 29887330 PMC6020856

[B47] Kostron H. Winkler H. Peer L. J. Konig P. (1977). Uptake of adenosine triphosphate by isolated adrenal chromaffin granules: a carrier-mediated transport. *Neuroscience* 2 159–166. 10.1016/0306-4522(77)90077-x 917274

[B48] Lalo U. Palygin O. Rasooli-Nejad S. Andrew J. Haydon P. G. Pankratov Y. (2014). Exocytosis of ATP from astrocytes modulates phasic and tonic inhibition in the neocortex. *PLoS Biol.* 12:e1001747. 10.1371/journal.pbio.1001747 24409095 PMC3883644

[B49] Larsson M. Sawada K. Morland C. Hiasa M. Ormel L. Moriyama Y. (2012). Functional and anatomical identification of a vesicular transporter mediating neuronal ATP release. *Cereb. Cortex* 22 1203–1214. 10.1093/cercor/bhr203 21810784

[B50] Lazarowski E. R. (2012). Vesicular and conductive mechanisms of nucleotide release. *Purinergic Signal.* 8 359–373. 10.1007/s11302-012-9304-9 22528679 PMC3360093

[B51] Lein E. S. Hawrylycz M. J. Ao N. Ayres M. Bensinger A. Bernard A. (2007). Genome-wide atlas of gene expression in the adult mouse brain. *Nature* 445 168–176. 10.1038/nature05453 17151600

[B52] Liu G. J. Kalous A. Werry E. L. Bennett M. R. (2006). Purine release from spinal cord microglia after elevation of calcium by glutamate. *Mol. Pharmacol.* 70 851–859. 10.1124/mol.105.021436 16760362

[B53] Liu H. T. Toychiev A. H. Takahashi N. Sabirov R. Z. Okada Y. (2008). Maxi-anion channel as a candidate pathway for osmosensitive ATP release from mouse astrocytes in primary culture. *Cell Res.* 18 558–565. 10.1038/cr.2008.49 18414449

[B54] Liu J. Liu W. Yang J. (2016). ATP-containing vesicles in stria vascular marginal cell cytoplasms in neonatal rat cochlea are lysosomes. *Sci. Rep.* 6:20903. 10.1038/srep20903 26864824 PMC4750035

[B55] Luqmani Y. A. (1981). Nucleotide uptake by isolated cholinergic synaptic vesicles: evidence for a carrier of adenosine 5′-triphosphate. *Neuroscience* 6 1011–1021. 10.1016/0306-4522(81)90067-1 7279210

[B56] Ma Y. Cao W. Wang L. Jiang J. Nie H. Wang B. (2014). Basal CD38/cyclic ADP-ribose-dependent signaling mediates ATP release and survival of microglia by modulating connexin 43 hemichannels. *Glia* 62 943–955. 10.1002/glia.22651 24578339

[B57] Masuda T. Ozono Y. Mikuriya S. Kohro Y. Tozaki-Saitoh H. Iwatsuki K. (2016). Dorsal horn neurons release extracellular ATP in a VNUT-dependent manner that underlies neuropathic pain. *Nat. Commun.* 7:12529. 10.1038/ncomms12529 27515581 PMC4990655

[B58] Menéndez Méndez A. (2017). *Caracterización Del Transportador Vesicular De Nucleótidos En Tejidos Neurales*. Ph.D. thesis, Complutense University of Madrid, Madrid.

[B59] Menendez-Mendez A. Diaz-Hernandez J. I. Miras-Portugal M. T. (2015). The vesicular nucleotide transporter (VNUT) is involved in the extracellular ATP effect on neuronal differentiation. *Purinergic Signal.* 11 239–249. 10.1007/s11302-015-9449-4 25847073 PMC4425722

[B60] Menendez-Mendez A. Diaz-Hernandez J. I. Ortega F. Gualix J. Gomez-Villafuertes R. Miras-Portugal M. T. (2017). Specific temporal distribution and subcellular localization of a functional vesicular nucleotide transporter (VNUT) in cerebellar granule neurons. *Front. Pharmacol.* 8:951. 10.3389/fphar.2017.00951 29311945 PMC5744399

[B61] Morin N. Di Paolo T. (2014). Interaction of adenosine receptors with other receptors from therapeutic perspective in Parkinson’s disease. *Int. Rev. Neurobiol.* 119 151–167. 10.1016/B978-0-12-801022-8.00007-6 25175965

[B62] Moriyama S. Hiasa M. (2016). Expression of vesicular nucleotide transporter in the mouse retina. *Biol. Pharm. Bull.* 39 564–569. 10.1248/bpb.b15-00872 27040629

[B63] Moriyama Y. Hiasa M. Sakamoto S. Omote H. Nomura M. (2017). Vesicular nucleotide transporter (VNUT): appearance of an actress on the stage of purinergic signaling. *Purinergic Signal.* 13 387–404. 10.1007/s11302-017-9568-1 28616712 PMC5563297

[B64] Moriyama Y. Nomura M. (2018). Clodronate: a vesicular ATP release blocker. *Trends Pharmacol. Sci.* 39 13–23. 10.1016/j.tips.2017.10.007 29146440

[B65] Muratore M. Quarta E. Grimaldi A. Calcagnile F. Quarta L. (2011). Clinical utility of clodronate in the prevention and management of osteoporosis in patients intolerant of oral bisphosphonates. *Drug Des. Devel. Ther.* 5 445–454. 10.2147/DDDT.S12139 22087064 PMC3210073

[B66] Nakagomi H. Yoshiyama M. Mochizuki T. Miyamoto T. Komatsu R. Imura Y. (2016). Urothelial ATP exocytosis: regulation of bladder compliance in the urine storage phase. *Sci. Rep.* 6:29761. 10.1038/srep29761 27412485 PMC4944198

[B67] Newman E. A. (2003). Glial cell inhibition of neurons by release of ATP. *J. Neurosci.* 23 1659–1666. 10.1523/jneurosci.23-05-01659.2003 12629170 PMC2322877

[B68] Nishida K. Nomura Y. Kawamori K. Moriyama Y. Nagasawa K. (2014). Expression profile of vesicular nucleotide transporter (VNUT, SLC17A9) in subpopulations of rat dorsal root ganglion neurons. *Neurosci. Lett.* 579 75–79. 10.1016/j.neulet.2014.07.017 25043192

[B69] Njus D. Kelley P. M. Harnadek G. J. (1986). Bioenergetics of secretory vesicles. *Biochim. Biophys. Acta* 853 237–265. 10.1016/0304-4173(87)90003-6 2887202

[B70] Omote H. Miyaji T. Hiasa M. Juge N. Moriyama Y. (2016). Structure, function, and drug interactions of neurotransmitter transporters in the postgenomic era. *Annu. Rev. Pharmacol. Toxicol.* 56 385–402. 10.1146/annurev-pharmtox-010814-124816 26514205

[B71] Omote H. Moriyama Y. (2013). Vesicular neurotransmitter transporters: an approach for studying transporters with purified proteins. *Physiology* 28 39–50. 10.1152/physiol.00033.2012 23280356

[B72] Orriss I. R. Knight G. E. Utting J. C. Taylor S. E. Burnstock G. Arnett T. R. (2009). Hypoxia stimulates vesicular ATP release from rat osteoblasts. *J. Cell. Physiol.* 220 155–162. 10.1002/jcp.21745 19259945

[B73] Oya M. Kitaguchi T. Yanagihara Y. Numano R. Kakeyama M. Ikematsu K. (2013). Vesicular nucleotide transporter is involved in ATP storage of secretory lysosomes in astrocytes. *Biochem. Biophys. Res. Commun.* 438 145–151. 10.1016/j.bbrc.2013.07.043 23876310

[B74] Pangrsic T. Potokar M. Stenovec M. Kreft M. Fabbretti E. Nistri A. (2007). Exocytotic release of ATP from cultured astrocytes. *J. Biol. Chem.* 282 28749–28758. 10.1074/jbc.M700290200 17627942

[B75] Pankratov Y. Lalo U. Verkhratsky A. North R. A. (2006). Vesicular release of ATP at central synapses. *Pflugers Arch.* 452 589–597. 10.1007/s00424-006-0061-x 16639550

[B76] Pankratov Y. Lalo U. Verkhratsky A. North R. A. (2007). Quantal release of ATP in mouse cortex. *J. Gen. Physiol.* 129 257–265. 10.1085/jgp.200609693 17325196 PMC2151610

[B77] Parpura V. Zorec R. (2010). Gliotransmission: exocytotic release from astrocytes. *Brain Res. Rev.* 63 83–92. 10.1016/j.brainresrev.2009.11.008 19948188 PMC2862866

[B78] Pascual O. Casper K. B. Kubera C. Zhang J. Revilla-Sanchez R. Sul J. Y. (2005). Astrocytic purinergic signaling coordinates synaptic networks. *Science* 310 113–116. 10.1126/science.1116916 16210541

[B79] Perez de Lara M. J. Guzman-Aranguez A. de la Villa P. Diaz-Hernandez J. I. Miras-Portugal M. T. Pintor J. (2015). Increased levels of extracellular ATP in glaucomatous retinas: possible role of the vesicular nucleotide transporter during the development of the pathology. *Mol. Vis.* 21 1060–1070. 26392744 PMC4558477

[B80] Pintor J. Diaz-Rey M. A. Torres M. Miras-Portugal M. T. (1992a). Presence of diadenosine polyphosphates–Ap4A and Ap5A–in rat brain synaptic terminals. Ca2+ dependent release evoked by 4-aminopyridine and veratridine. *Neurosci. Lett.* 136 141–144. 10.1016/0304-3940(92)90034-5 1641181

[B81] Pintor J. Kowalewski H. J. Torres M. Mirasportugal M. T. Zimmermann H. (1992b). Synaptic vesicle storage of diadenosine polyphosphates in the torpedo electric organ. *Neurosci. Res. Commun.* 10 9–15.

[B82] Pintor J. Rotllan P. Torres M. Miras-Portugal M. T. (1992c). Characterization and quantification of diadenosine hexaphosphate in chromaffin cells: granular storage and secretagogue-induced release. *Anal. Biochem.* 200 296–300. 10.1016/0003-2697(92)90469-n 1632493

[B83] Reimer R. J. (2013). SLC17: a functionally diverse family of organic anion transporters. *Mol. Aspects Med.* 34 350–359. 10.1016/j.mam.2012.05.004 23506876 PMC3927456

[B84] Richardson P. J. Brown S. J. (1987). ATP release from affinity-purified rat cholinergic nerve terminals. *J. Neurochem.* 48 622–630. 10.1111/j.1471-4159.1987.tb04138.x 2432187

[B85] Rodriguez del Castillo A. Torres M. Delicado E. G. Miras-Portugal M. T. (1988). Subcellular distribution studies of diadenosine polyphosphates–Ap4A and Ap5A–in bovine adrenal medulla: presence in chromaffin granules. *J. Neurochem.* 51 1696–1703. 10.1111/j.1471-4159.1988.tb01147.x 2846780

[B86] Roseth S. Fykse E. M. Fonnum F. (1995). Uptake of L-glutamate into rat brain synaptic vesicles: effect of inhibitors that bind specifically to the glutamate transporter. *J. Neurochem.* 65 96–103. 10.1046/j.1471-4159.1995.65010096.x 7790899

[B87] Sakamoto S. Miyaji T. Hiasa M. Ichikawa R. Uematsu A. Iwatsuki K. (2014). Impairment of vesicular ATP release affects glucose metabolism and increases insulin sensitivity. *Sci. Rep.* 4:6689. 10.1038/srep06689 25331291 PMC4204045

[B88] Sawada K. Echigo N. Juge N. Miyaji T. Otsuka M. Omote H. (2008). Identification of a vesicular nucleotide transporter. *Proc. Natl. Acad. Sci. U.S.A.* 105 5683–5686. 10.1073/pnas.0800141105 18375752 PMC2311367

[B89] Sawynok J. Downie J. W. Reid A. R. Cahill C. M. White T. D. (1993). ATP release from dorsal spinal cord synaptosomes: characterization and neuronal origin. *Brain Res.* 610 32–38. 10.1016/0006-8993(93)91213-c 8518929

[B90] Shinozaki Y. Nomura M. Iwatsuki K. Moriyama Y. Gachet C. Koizumi S. (2014). Microglia trigger astrocyte-mediated neuroprotection via purinergic gliotransmission. *Sci. Rep.* 4:4329. 10.1038/srep04329 24710318 PMC3948352

[B91] Sottile V. Li M. Scotting P. J. (2006). Stem cell marker expression in the bergmann glia population of the adult mouse brain. *Brain Res.* 1099 8–17. 10.1016/j.brainres.2006.04.127 16797497

[B92] Stout C. E. Costantin J. L. Naus C. C. Charles A. C. (2002). Intercellular calcium signaling in astrocytes via ATP release through connexin hemichannels. *J. Biol. Chem.* 277 10482–10488. 10.1074/jbc.M109902200 11790776

[B93] Suadicani S. O. Brosnan C. F. Scemes E. (2006). P2X7 receptors mediate ATP release and amplification of astrocytic intercellular Ca2+ signaling. *J. Neurosci.* 26 1378–1385. 10.1523/JNEUROSCI.3902-05.2006 16452661 PMC2586295

[B94] Suadicani S. O. Iglesias R. Wang J. Dahl G. Spray D. C. Scemes E. (2012). ATP signaling is deficient in cultured Pannexin1-null mouse astrocytes. *Glia* 60 1106–1116. 10.1002/glia.22338 22499153 PMC3348971

[B95] Tompkins J. D. Parsons R. L. (2006). Exocytotic release of ATP and activation of P2X receptors in dissociated guinea pig stellate neurons. *Am. J. Physiol. Cell Physiol.* 291 C1062–C1071. 10.1152/ajpcell.00472.2005 16760262

[B96] Tozaki-Saitoh H. Tsuda M. Miyata H. Ueda K. Kohsaka S. Inoue K. (2008). P2Y12 receptors in spinal microglia are required for neuropathic pain after peripheral nerve injury. *J. Neurosci.* 28 4949–4956. 10.1523/JNEUROSCI.0323-08.2008 18463248 PMC6670742

[B97] Tsuda M. Shigemoto-Mogami Y. Koizumi S. Mizokoshi A. Kohsaka S. Salter M. W. (2003). P2X4 receptors induced in spinal microglia gate tactile allodynia after nerve injury. *Nature* 424 778–783. 10.1038/nature01786 12917686

[B98] Verderio C. Cagnoli C. Bergami M. Francolini M. Schenk U. Colombo A. (2012). TI-VAMP/VAMP7 is the SNARE of secretory lysosomes contributing to ATP secretion from astrocytes. *Biol. Cell.* 104 213–228. 10.1111/boc.201100070 22188132

[B99] Vessey K. A. Fletcher E. L. (2012). Rod and cone pathway signalling is altered in the P2X7 receptor knock out mouse. *PLoS One* 7:e29990. 10.1371/journal.pone.0029990 22253851 PMC3254638

[B100] Weber A. Winkler H. (1981). Specificity and mechanism of nucleotide uptake by adrenal chromaffin granules. *Neuroscience* 6 2269–2276. 10.1016/0306-4522(81)90016-6 7329547

[B101] Whittaker V. P. (1987). Cholinergic synaptic vesicles from the electromotor nerve terminals of torpedo. Composition and life cycle. *Ann. N. Y. Acad. Sci.* 493 77–91. 10.1111/j.1749-6632.1987.tb27185.x 3296914

[B102] Winkler H. (1976). The composition of adrenal chromaffin granules: an assessment of controversial results. *Neuroscience* 1 65–80. 10.1016/0306-4522(76)90001-4 794758

[B103] Wise R. A. (2008). Dopamine and reward: the anhedonia hypothesis 30 years on. *Neurotox. Res.* 14 169–183. 10.1007/BF03033808 19073424 PMC3155128

[B104] Zhang J. M. Wang H. K. Ye C. Q. Ge W. Chen Y. Jiang Z. L. (2003). ATP released by astrocytes mediates glutamatergic activity-dependent heterosynaptic suppression. *Neuron* 40 971–982. 10.1016/s0896-6273(03)00717-7 14659095

[B105] Zhang Z. Chen G. Zhou W. Song A. Xu T. Luo Q. (2007). Regulated ATP release from astrocytes through lysosome exocytosis. *Nat. Cell Biol.* 9 945–953. 10.1038/ncb1620 17618272

